# Optimizing the Catalytic Performance of Ba_1−x_Ce_x_MnO_3_ and Ba_1−x_La_x_Cu_0.3_Mn_0.7_O_3_ Perovskites for Soot Oxidation in Simulated GDI Exhaust Conditions

**DOI:** 10.3390/molecules29133190

**Published:** 2024-07-04

**Authors:** Nawel Ghezali, Álvaro Díaz-Verde, María José Illán-Gómez

**Affiliations:** Carbon Materials and Envriroment Research (MCMA) Group, Inorganic Chemistry Department, Institute of Materials of the University of Alicante (IUMA), Faculty of Sciences, University of Alicante, 03690 Alicante, Spain; ghezalinawel34@gmail.com (N.G.); alvaro.diaz@ua.es (Á.D.-V.)

**Keywords:** sol–gel, perovskites, cerium, lanthanum, soot oxidation, GDI

## Abstract

Ba_1−x_Ce_x_MnO_3_ (BM-Ce_x_) and Ba_1−x_La_x_Mn_0.7_Cu_0.3_O_3_ (BMC-La_x_) perovskite-type mixed oxides were synthesized using the sol–gel method adapted for aqueous media with different values of x (0, 0.1, 0.3, 0.6) to estimate the effect of the degree of the partial substitution of Ba by Ce or La on the structure and properties that are relevant for their use as catalysts for gasoline direct injection (GDI) soot oxidation. The samples were deeply characterized by ICP-OES, XRD, XPS, N_2_ adsorption, H_2_-TPR, and O_2_-TPD, and their potential as catalysts for soot oxidation has been analyzed in various scenarios that replicate the exhaust conditions of a GDI engine. By comparing the catalytic performance for soot oxidation of the two tested series (BM-Cex and BMC-Lax) and in the two conditions used (100% He and 1% O_2_ in He), it could be concluded that (i) in the absence of oxygen in the reaction atmosphere (100% He), BMC-La_0.1_ is the best catalyst, as copper is also able to catalyze the soot oxidation; and (ii) if oxygen is present in the reaction atmosphere (1% O_2_/He), BM-Ce_0.1_ is the most-active catalyst as it presents a higher proportion of Mn(IV) than BMC-La_0.1_. Thus, it seems that the addition of an amount of Ce or La higher than that corresponding to x = 0.1 in Ba_1−x_Ce_x_MnO_3_ and Ba_1−x_La_x_Cu_0.3_Mn_0.7_O_3_ does not allow us to improve the catalytic performance of BM-Ce_0.1_ and BMC-La_0.1_ for soot oxidation in the tested conditions.

## 1. Introduction

Perovskite-type mixed oxides (with the general formula ABO_3_) are becoming increasingly popular in the materials science and catalysis fields due to their tunable properties [1,2,3,4]. The unique crystal structure, the non-stoichiometry in oxygen, the acid–base and redox properties, and the thermal stability enable perovskites to be effective as catalysts for different reactions, including, among others, oxidation, hydrogenation, and photocatalytic reactions [1,2,3,4]. Moreover, perovskites are also being employed for electrocatalytic applications as solid oxide fuel cells due to their oxygen-ion-conducting properties [5]. Considering the main topic of the present work, perovskite-type oxides are being used as catalysts in the process related to the post-treatment of car exhaust gases, such as carbon monoxide, hydrocarbon and/or soot oxidation, and NO_x_ reduction [1,3,4,6]. Focusing our attention on soot oxidation, this application has become relevant in recent years as GDI engines are considered as a novel option which ensures a higher fuel economy and lower pollutant emissions compared with the traditional port fuel injection engines [7]. Regarding perovskites, it is generally accepted that their catalytic activity for soot oxidation is related to the presence of oxygen vacancies, to the oxygen’s mobility through the solid, and to the redox properties [1,4]. Additionally, it has been proved that modifications on some physical properties of perovskites, such as particle size, surface area, morphology, and crystal structure, as well as composition, affect the catalytic performance [6,8]. Considering the composition of perovskites, it has been demonstrated that the incorporation of cerium and lanthanum in the A-site of ABO_3_ perovskites improves the catalytic performance of these oxides by promoting the creation of oxygen vacancies and by enhancing the redox properties, and thus the mobility, of oxygen [9,10,11,12,13,14,15]. In particular, some perovskites based on Ba_0.9_A_0.1_MnO_3_ and Ba_0.9_A_0.1_Mn_0.7_Cu_0.3_O_3_ (A = Mg, Ce, La, Ca, Sr) formulations have been previously employed as catalysts for soot oxidation in simulated GDI engine exhaust conditions [16], and researchers have concluded that Ba_0.9_Ce_0.1_MnO_3_ is the most active in 1% O_2_/He (being T_50%_ = 641 °C, which is the temperature for achieving 50% of soot oxidation versus 710 °C for BaMnO_3_ raw perovskite), while Ba_0.9_La_0.1_Cu_0.3_Mn_0.7_O_3_ presents the best performance in the absence of oxygen (T_10%_ = 611 °C, which is the temperature for achieving 10% of soot oxidation versus 879 °C for BaCu_0.3_Mn_0.7_O_3_ perovskite). However, as the content of cerium or lanthanum could be relevant for the catalytic performance [17], the goal of this work is to evaluate the influence of the degree of cerium and lanthanum substitution on the properties of Ba_1−x_Ce_x_MnO_3_ (denoted as BM-Cex) and Ba_1−x_La_x_Cu_0.3_Mn_0.7_O_3_ (named as BMC-La_x_) (x = 0, 0.1, 0.3, 0.6) perovskite-type mixed oxides that determine their use as catalysts for GDI soot oxidation.

## 2. Results and Discussion

### 2.1. Catalysts Characterization

#### 2.1.1. Ba_1−x_Ce_x_MnO_3_ (x = 0, 0.1, 0.3, 0.6)

Table 1 shows the nomenclature and the actual cerium content (wt%) determined using Inductively Coupled Plasma Optical Emission Spectroscopy (ICP-OES) and the Brunauer–Emmett–Teller (BET) surface area. As a reference, the data corresponding to the raw BaMnO_3_ (BM) perovskite have been included.

First, as expected, the cerium percentage increases with the degree of substitution, ranging from 1.3 wt% to 6.0 wt%. The samples present low BET surface areas, as is usual for perovskite-type mixed oxides [1,18,19], because the porosity was not well developed during the synthesis, probably due to the relatively high calcination temperature used (850 °C) [20,21].

**Figure 1:**
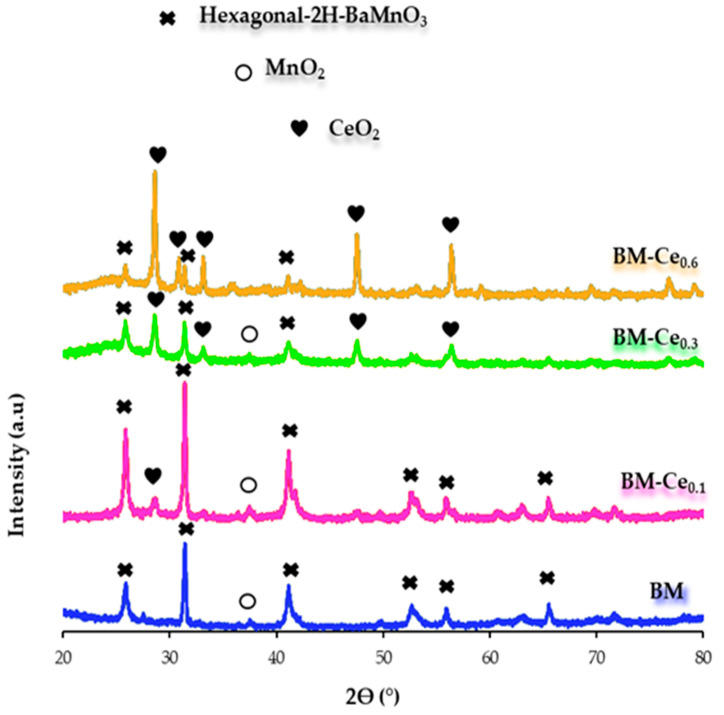
XRD patterns of BM-Cex samples.

In the X-ray diffraction (XRD) patterns of BM-Cex perovskites (Figure 1), diffraction peaks at c.a. 2θ 25.8°, 31.4°, 41.1°, 52.8°, 55.9°, and 65.7°, corresponding to the hexagonal 2H-BaMnO_3_ perovskite structure (PDF number: 026-0168, designated by the International Centre of Diffraction Data, ICDD), are identified. As the percentage of Ce in the composition of perovskites increases, the intensity of the peaks corresponding to the hexagonal perovskite structure decreases, being replaced by peaks at c.a. 28.6°, 33.2°, 47.5°, and 56.5° 2θ values (PDF numbers: 43-1002) corresponding to CeO_2_. In fact, for BM-Ce_0.6_, this phase becomes the main one, while it is a minority phase for BM-Ce_0.1_. On the other hand, only MnO_2_ (PDF numbers: 024-0735) was detected as a minority phase for BM, BM-Ce_0.1_, and BM-Ce_0.3_, being the relatively low calcination temperature used during the sol–gel synthesis (850 °C), which is probably responsible for its presence. Thus, the intensity of the main XRD peak for the hexagonal perovskite structure increases for BM-Ce_0.1_ with respect to BM. However, at higher values of x, the intensity decreases in favor of the CeO_2_ crystalline phase, which increases until becoming the most intense peak for BM-Ce_0.6_. The average crystallite size displayed in Table 2 was calculated by applying the Williamson–Hall method to the XRD peaks of hexagonal-2H-BaMnO_3_ phase [22], and a decrease is observed in the presence of cerium in favor of CeO_2_ phase. Finally, the values calculated for “a” and “c” cell parameters for the hexagonal structure, as depicted in Table 2, closely resemble those of the reference sample (BM); so, it seems that the incorporation of Ce into the perovskite network does not modify these parameters, as cerium appears as CeO_2_, avoiding the modification of the BaMnO_3_ structure. The described trend of the different crystalline phases with respect to the Ce percentage is confirmed by the XRD refinement data shown in Table 2.

**Figure 2:**
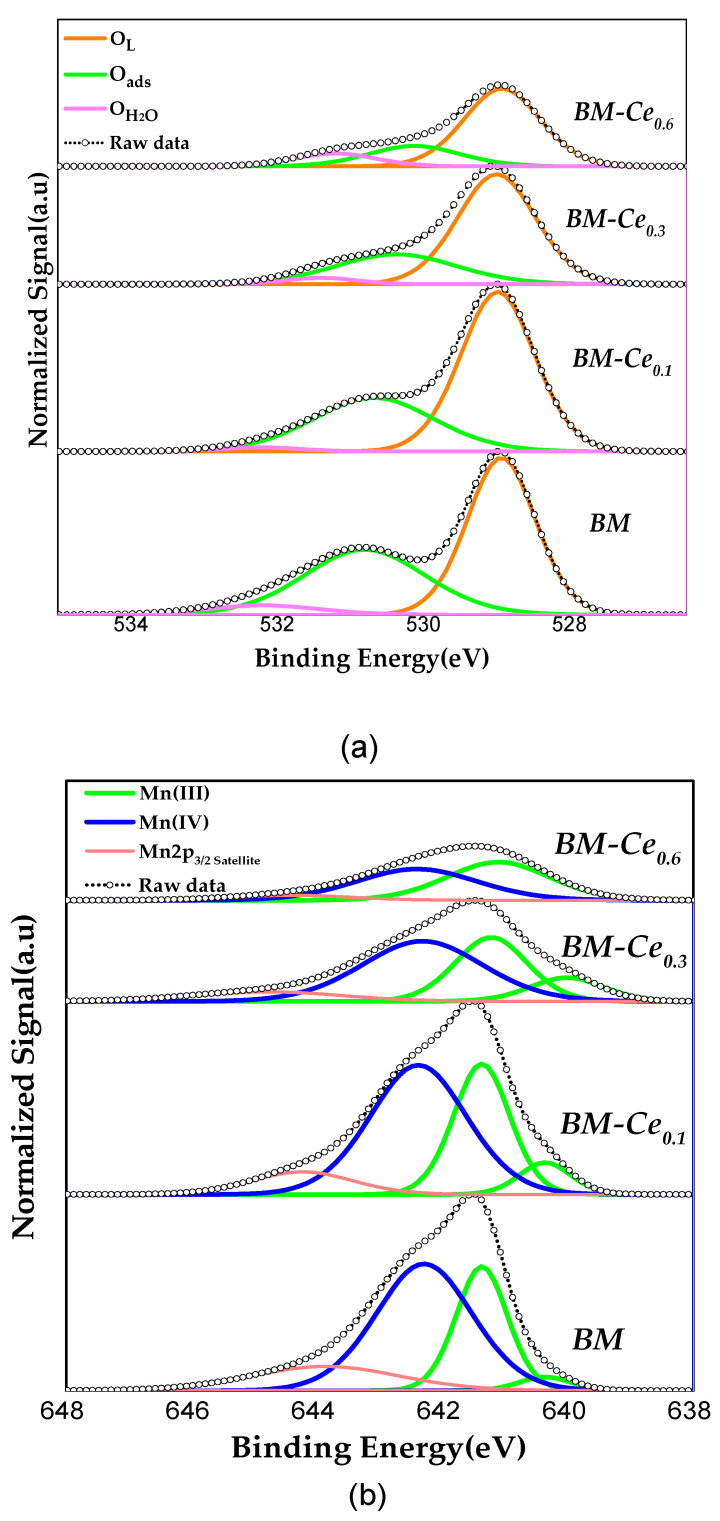

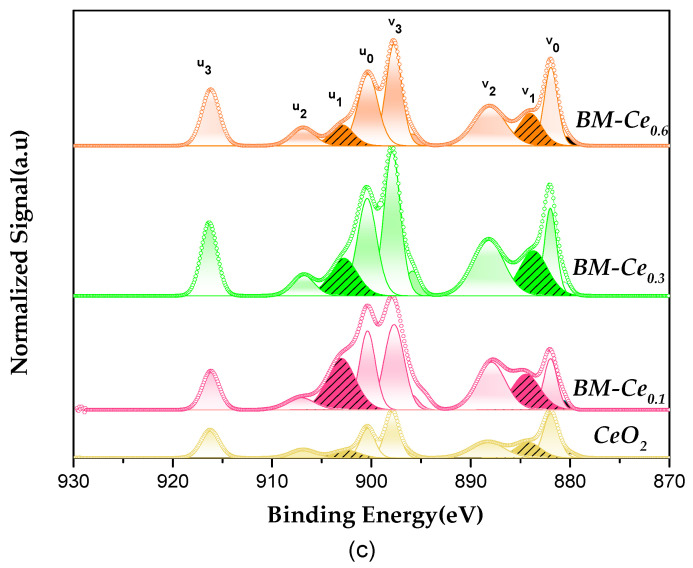
XPS spectra of BM-Cex samples for the (**a**) O 1s, (**b**) Mn 2p_3/2_, and (**c**) Ce 3d core-level regions.

To determine the surface composition and oxidation states of the elements present on the surface of samples, the X-ray photoelectron spectroscopy (XPS) technique was employed (see the Materials and Methods section for details), and the most relevant findings are shown in Figure 2 and Table 3. The XPS spectra for the O 1s transition (Figure 2a) show three peaks at c.a. 529.1, 531.1, and 532.7 eV, attributable to lattice oxygen (O_L_), absorbed oxygen species (O_ads_) that include surface carbonate (CO_3_^−2^), hydroxyl groups (OH^−^), peroxide (O_2_^−2^) or superoxide (O^−2^) ions and defect sites with low oxygen coordination (oxygen vacancies), and, finally, adsorbed water species (O_H2O_) [23,24,25]. By comparing with raw BM perovskite, the binding energy (BE) corresponding to the maximum of O_ads_ peaks shifted towards lower values with the increase in the cerium content due to the presence of more oxygen species (from CeO_2_) on the surface [26], while no significant shift is detected in BE max for O_L_. As the O_L_/(Ba + Ce + Mn) ratio (calculated using the area under the O_L_ peak and the area of the peaks for cations present on surface) is lower than the theoretical value for perovskites (1.5), oxygen vacancies are present on the surface of all samples, and, as the values rise from 1 to 1.2, an increase in the amount of lattice oxygen species occurs, which seems to be due to the presence of the CeO_2_.

According to the literature, the assignment of the manganese oxidation states via XPS is a difficult task [27,28,29,30,31]; however, the raw data for Mn 2p_3/2_ spectra suggest the presence of Mn (III) and Mn (IV). Considering the asymmetric shape of the XPS signal, and based on the analysis of Ponce et al. for manganese-based perovskites [32], the two components of the Mn 2p_3/2_ spectra (Figure 2b) could be interpreted as follows: the deconvoluted peak at BE = 641.4–640.6 eV corresponds to Mn(III), and the observed peak at BE = 642.5–642.1 eV to Mn(IV). The presence of the satellite peak of Mn(III) at a binding energy of c.a. 644 eV allows us to confirm that Mn(III) exists on the surface. For BM-Ce_0.3_ and BM-Ce_0.6_ samples, a slight shift towards lower binding energies of Mn(III) with respect to BM and BM-Ce_0.1_ peak can be observed, which should be related to the increased amount of CeO_2_ detected via XRD. Thus, these spectra reveal that Mn(III) and Mn(IV) coexist on the surface of all perovskites [30,31,33], as these oxidation states allow for the surface to become electroneutral. Note also that for most of samples, Mn(III) shows two deconvolutions that reveal two different electronic environments, probably originating in the presence of oxygen vacancies and/or in the existence of Ce in different oxidation states, as it will be discussed below. The Mn(IV)/Mn(III) ratio values (calculated using the area under the corresponding deconvoluted XPS peaks and summarized in Table 3) decrease as the cerium percentage increases; so, the presence of cerium promotes an increase in Mn(III) on the surface with respect to Mn(IV), with this fact also being related to the electroneutrality (as Ce(IV)/Ce(III) is replacing Ba(II) in the perovskite formulation).Thus, Mn(IV) is the main oxidation state on the surface for BM and BM-Ce_0.1_, but it is Mn(III) for the other two samples with higher cerium percentages. Note that BM-Ce_0.6_ does not follow this trend, as the Mn(IV)/Mn(III) ratio increases with respect to BM-Ce_0.3_. So, as the ratio is close to 1, the surface of this sample should present an almost-similar amount of both oxidation states.

Figure 2c illustrates the XPS spectra corresponding to the Ce 3d orbitals. According to relevant literature [34,35,36,37], the deconvolution of these spectra reveals eight sub-peaks: four v quadruplets within the Ce 3d_5/2_ region and four u quadruplets within the Ce 3d_3/2_ region. Within these sub-peaks, the Ce(III) oxidation state is discernible through the two specific subpeaks v_1_ and u_1_, being the remaining sub-peaks associated with the Ce(IV) oxidation state. Thus, both Ce(III) and Ce(IV) are present on the surface of all BM-Ce_x_ samples, but as the Ce(IV)/Ce(III) ratios are higher than 1, this means that the amount of Ce(IV) is higher than that of Ce(III). The presence of the two valences of Ce on the surface seems to be relevant as it could enhance the oxygen storage capacity [9]. Moreover, as the Ce(IV)/Ce(III) ratio increases with the Ce percentage, the Ce(IV) proportion also increases from BM-Ce_0.1_ to BM-Ce_0.6_. This finding agrees with the XRD data, which reveal the prevalence of CeO_2_ in the bulk of samples as Ce content increases.

**Figure 3:**
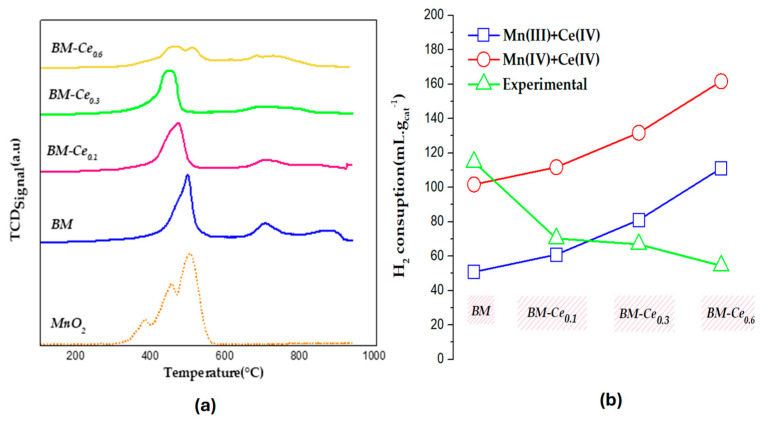
(**a**) H_2_-TPR profiles of BM, BM-Cex samples, and MnO_2_ as a reference; (**b**) H_2_ consumption (mL g^−1^ of catalyst).

Temperature-programmed reduction tests with H_2_ (H_2_-TPR) were used to evaluate the reducibility of BM-Cex mixed oxides, as the reducibility is related to its activity in the oxidation process [38]. Figure 3a shows the hydrogen consumption profiles of BM and BM-Cex perovskites, as well as the corresponding MnO_2_ used as a reference. The H_2_-TPR profile for MnO_2_ shows two main reduction peaks with maxima at around 400 and 500 °C. The first main reduction peak (at ∼400 °C) is associated with the reduction of MnO_2_ and/or Mn_2_O_3_ to Mn_3_O_4_; whereas the second main peak (at ∼500 °C) is assigned to the reduction of Mn_3_O_4_ to MnO [38]. The H_2_-TPR profiles of BM and all BM-Cex samples exhibit three reduction peaks: (i) the first one, in the temperature region between around 300 and 600 °C, corresponds to the reduction of Mn(IV)/Mn(III) to Mn(II), as well as, for Ce-doped samples, to the reduction of surface Ce(IV) to Ce(III) [31,36]; (ii) in the intermediate temperature region (between 600 and 800 °C), the observed peak is linked to the desorption/reduction of oxygen species and to the reduction of the CeO_2_ bulk phase [39] for cerium-doped samples; and, finally, (iii) the very-low-intensity peak at the highest temperature region (between 900–1000 °C) is associated with the reduction of bulk Mn(III). After doping with Ce, the main reduction peak (with a maximum at around 500 °C) is slightly shifted towards lower temperatures with respect to the undoped BM perovskite (482 °C versus 468, 442, and 457 °C for BM-Ce_0.1_, BM-Ce_0.3_, and BM-Ce_0.6_, respectively); so, the reduction of Mn/Ce takes place at a lower temperature for the doped samples, probably due to a synergetic effect between Mn and Ce [40]. The experimental hydrogen consumption per gram of catalyst, calculated between 200 °C and 600 °C (using the hydrogen consumption patterns illustrated in Figure 3a, is compared in Figure 3b with the hydrogen consumption calculated, assuming the entire reduction of Ce (IV) and Mn as either Mn(III) (blue line) or Mn(IV) (red line). For BM, it seems that Mn(IV) is the main oxidation state in the bulk, as the experimental value is close to the corresponding point in the red line. However, after doping with cerium, the experimental H_2_ consumptions approach Mn(III) (and Ce(IV)), so Mn(III) seems to be the main oxidation state in the bulk of the BM-Cex samples. However, a decrease is observed from BM-Ce_0.1_ to BM-Ce_0.6_, probably due to the increasing amount of bulk CeO_2_ (as detected via XRD).

**Figure 4:**
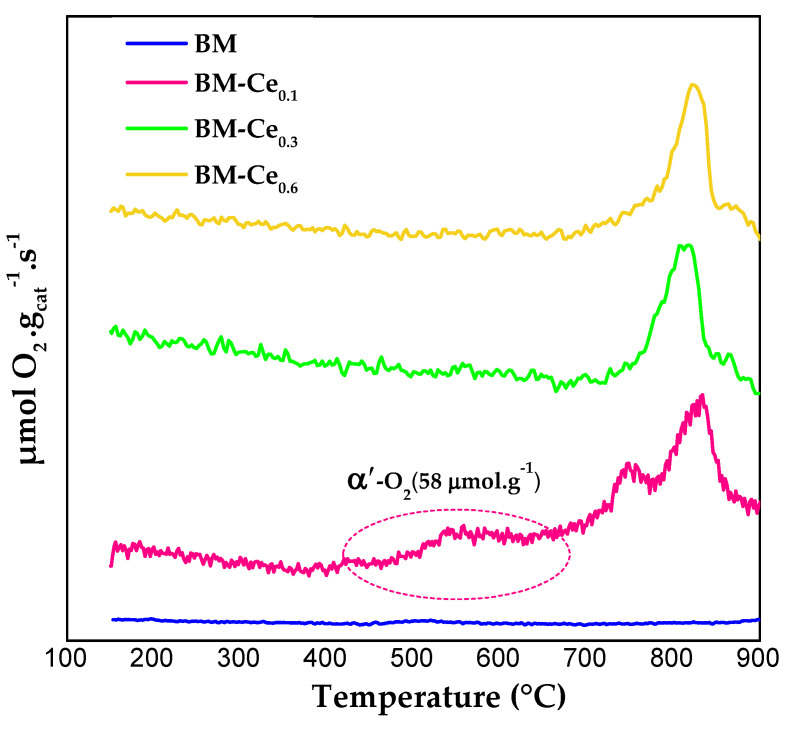
O_2_-TPD profiles for BM and BM-Cex samples.

The mobility of O_2_ for BM-Cex samples was investigated by analyzing the emission of O_2_ during the temperature-programmed desorption of O_2_ (O_2_-TPD) tests, and the O_2_ evolution profiles are shown in Figure 4. The amount of O_2_ released from perovskites was determined by the integration of the O_2_ desorption profiles, using CuO as a reference [31] and assuming that CuO is converted to Cu_2_O [41,42]. For perovskite-type mixed oxides, three types of oxygen are usually identified: α-O_2_, α’-O_2_, and β-O_2_. α-O_2_ refers to oxygen that evolves from that adsorbed on the surface vacancies; α′-O_2_ corresponds to oxygen coming from that the adsorbed on the surface lattice defects, such as dislocations or grain borders; and finally, β-O_2_ is the oxygen desorbed from the perovskite lattice [1,27,30], which is dependent on the partial reduction of Mn(IV) to Mn(III) [43,44], and also on the reduction of Ce(IV) to Ce(III) for BM-Cex samples [12], and whose desorption is facilitated by the presence of bulk oxygen vacancies. All BM-Cex samples evolve oxygen in the high-temperature region (β-O_2_), and the amount evolved (calculated from the area under the O_2_ profiles and using CuO as reference) increases from 114 μmol g^−1^ for BM-Ce_0.1_ to 136 μmol g^−1^ for BM-Ce_0.3_, but it decreases to 98 μmol g^−1^ for BM-Ce_0.6_. So, the oxygen evolved is notably higher for BM-Cex samples (as BM only generates 23 μmol g^−1^), revealing an improved oxygen mobility through the perovskite lattice in the presence of Ce, probably due to the contribution of the Ce(IV)/Ce(III) redox pair. However, this trend is not followed by BM-Ce_0.6_, and this finding seems to be related to the decrease in the reducibility as the Ce content increases, as revealed by the H_2_-TPR results (Figure 3). Finally, the BM-Ce_0.1_ sample shows a small desorption peak between 500 and 650 °C, which corresponds to the oxygen adsorbed on the surface lattice defects (α′-O_2_), which is highlighted in Figure 4.

#### 2.1.2. Ba_1−x_La_x_Mn_0.7_Cu_0.3_O_3_ (x = 0, 0.1, 0.3, 0.6)

Table 4 lists the nomenclature, the La and Cu metal contents (obtained by ICP-OES), and the BET surface area of Ba_1−x_La_x_Mn_0.7_Cu_0.3_O_3_ (BMC-La_x_) samples. According to the ICP-OES data, the percentage of Cu ranges from 7.1 wt% for BMC-La_0.6_ to 9.8 wt% for BMC-La_0.3_, showing a wt% La from 5.4 wt% to 24.0 wt%. As seen in Table 4, the BET surface area of perovskites is lower than 10 m^2^ g^−1^, and these low surface areas are consistent with what is expected for solids with very low developed porosity such as perovskite-type mixed oxides [18,19]. As indicated above, this is most likely due to the relatively high calcination temperature used during synthesis (850 °C) [20,21].

**Figure 5:**
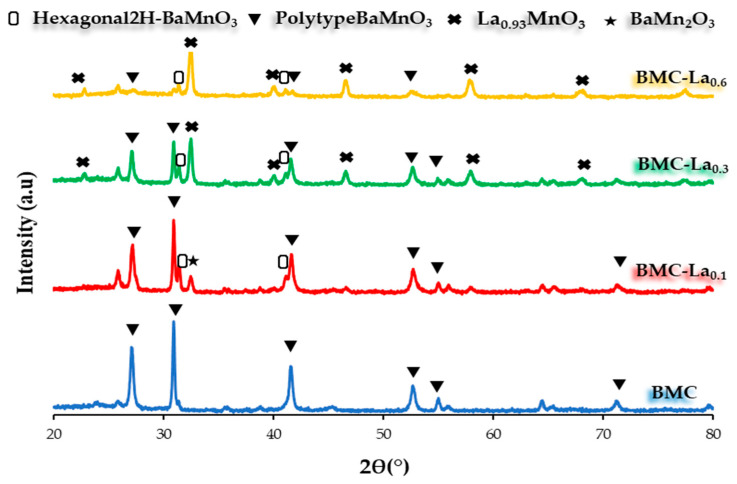
XRD patterns of the BMC and BMC-Lax samples.

The XRD patterns obtained for BMC-Lax samples are displayed in Figure 5, along with the BaMn_0.7_Cu_0.3_O_3_ (BMC) perovskite used as a reference, and Table 5 contains the most important related data. All the identified peaks belong to a perovskite structure, and only for BMC-La_0.1_ is a very small intensity peak corresponding to BaMn_2_O_3_ (PDF number: 01-071-1325) detected as a minority phase. In addition, it is observed that as the percentage of lanthanum increases, the intensity of the XRD peaks corresponding to the polytype BaMnO_3_ perovskite structure [21]—at 27.0°, 30.9°, 27.5°, 41.5°, 52.9°, 54.8°, 64.3°, and 71.0°—decreases in favor of an increase in the intensity of the peaks corresponding to hexagonal 2H-BaMnO_3_ (PDF number: 026-0168) and La_0.93_MnO_3_ trigonal perovskites structures at 31.5°/41.3° and 23.1°/32.6°/40.2°/46.6/58.1°, respectively (PDF number: 01-082-1152). Note that the XRD peaks of La_0.93_MnO_3_ phase are present with similar or higher intensity with respect to those of the polytype BaMnO_3_ phase for the two samples with high lanthanum content; so, as the La percentage increases, the formation of this La-based perovskite is favored, becoming the main phase for BMC-La_0.6_. So, if the degree of substitution of Ba by La is higher than that corresponding to x = 0.1, the insertion of La seems to be more difficult. The coexistence of the hexagonal and polytype perovskite structures for low contents of lanthanum-based perovskite indicates that the presence of La in the lattice hinders the incorporation of copper, which causes the change from the polytype hexagonal structure [16,21]. The average crystal sizes of the polytype BaMnO_3_ phase, calculated by applying the Williamson–Hall method to the corresponding XRD peaks [22], and reported in Table 5, indicate that the addition of lanthanum reduces the average crystal size of the polytype structure, but, when the trigonal structure becomes relevant (so for BMC-La_0.3_ and BMC-La_0.6_), the trend changes, and the average crystal size increases with the lanthanum content. On the contrary, the lattice parameters, corresponding to the polytype structure for BMC and BMC-La_0.1_ and to the trigonal structure for BMC-La_0.3_ and BMC-La_0.6_, do not appreciably change with the La percentage. As for the BM-Cex series, the XRD refinement data, included in Table 5, confirm the above-described trend of the different crystalline phases with the increase in the La percentage.

**Figure 6:**
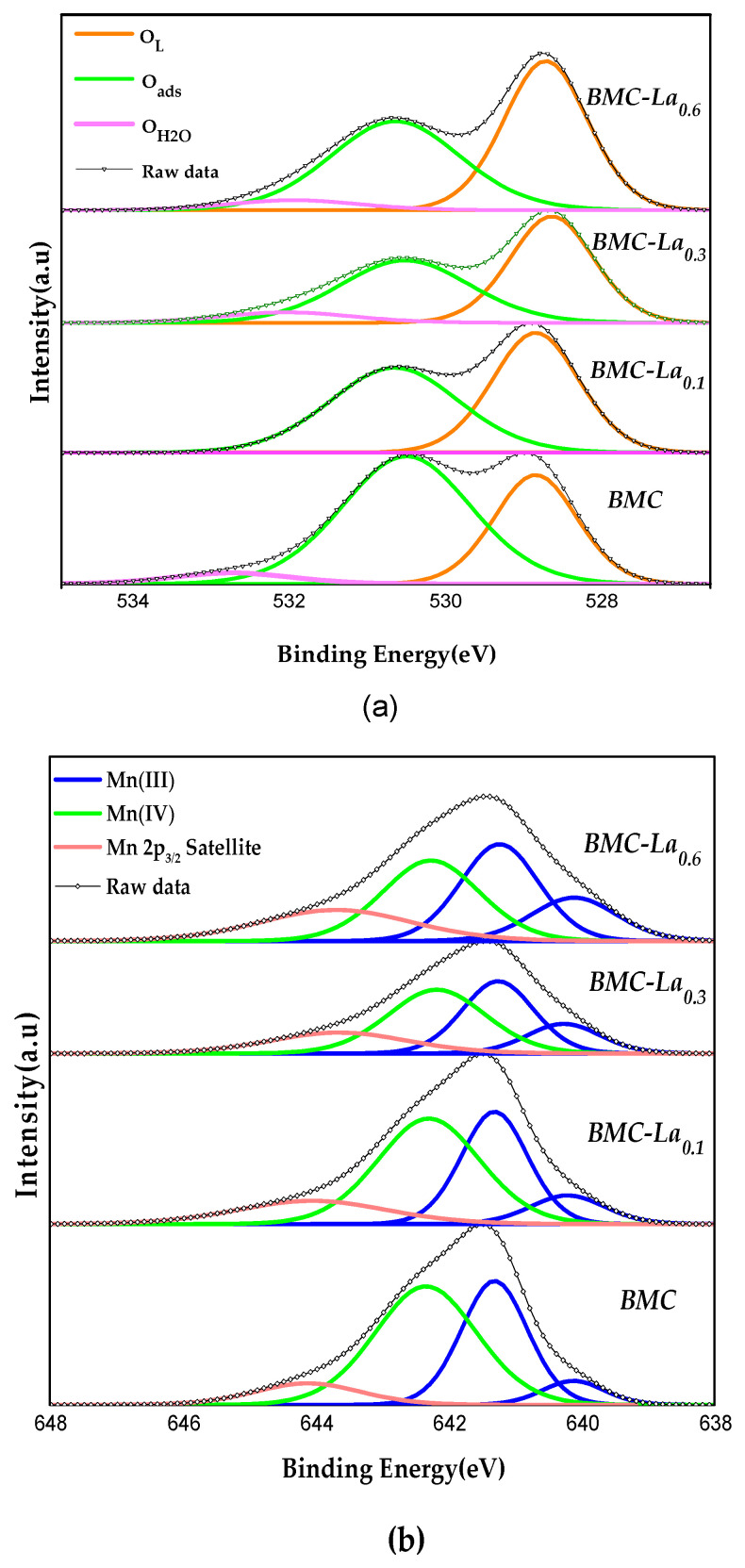

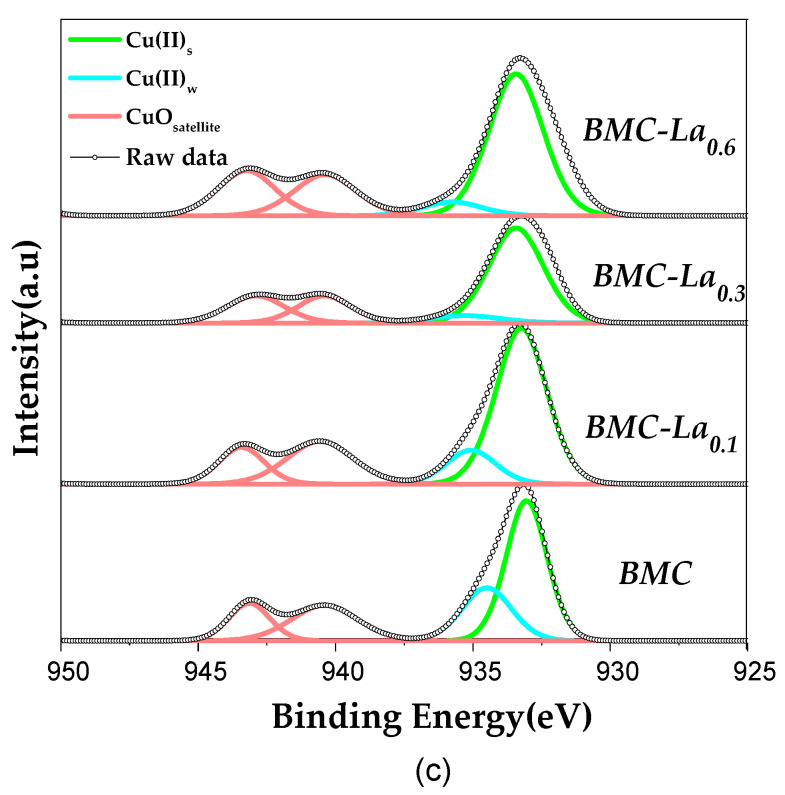
XPS spectra of BMC and BMC-Lax samples in the (**a**) O1s, (**b**) Mn2p_3/2_, and (**c**) Cu 2p_3/2_ core-level regions.

Information regarding the surface composition and the oxidation state of the elements present on the surface (up to around 5 nm in depth) is obtained using the XPS technique. The spectra for O 1s, Mn 2p_3/2_, and Cu2p_3/2_ transitions are displayed in Figure 6.

For perovskites, the O 1s spectrum (Figure 6a) could be deconvoluted into three peaks with maximum binding energies at around 528.5, 530.5, and 533.0 eV that correspond to [23,24,25]: (i) lattice oxygen (O_L_); (ii) adsorbed oxygen species (named “O_ads_”, which includes surface carbonate (CO_3_^2−^), hydroxyl groups (OH^−^), peroxide (O_2_^2−^) or superoxide (O^2−^) ions, and oxygen defect sites); and (iii) chemisorbed water (O_H2O_). After the addition of La, the area under the different deconvoluted peaks seems to have been modified. To analyze the effect of the different amounts of La in the lattice oxygen, the values of the O_L_/(Ba + Mn + La) ratio have been calculated using the area under the O_L_ peak and the area of the peaks for the cations present on the surface. As all experimental values included in Table 6 are lower than the corresponding nominal value for ABO_3_ (1.5), this means that, as observed for BM-Cex samples, oxygen vacancies exist on the surface [23,24] of all perovskites. These vacancies are generated to achieve the neutrality of positive and negative charges on the surface of the samples because a defect in the positive charge exists due to the presence of Cu (II) and the coexistence of Mn(III) and Mn(IV) (see discussion below). Note that after the addition of La, the value only increases from 0.8 to 0.9, and it does not further change with the La percentage. So, the amount of lattice oxygen, and consequently, of oxygen vacancies, seems not depending on the increase in the La percentage in the samples.

As was indicated above for the BM-Cex series, the assignment of the manganese oxidation states via XPS is a difficult task [25,44,45]; however, the raw data for the Mn 2p_3/2_ spectra suggest their presence on the surface of Mn (III) and Mn (IV). Following the same pathway as for the Ce series (the asymmetric shape of the XPS signal and based on the Ponce et al. criteria for manganese-based perovskites [32]), the Mn 2p_3/2_ spectra (Figure 6b) could be interpreted as follows: (i) the signal at the highest binding energy corresponds to the Mn (III) satellite peak; (ii) the peak at intermediated binding energy (around 642 eV) is assigned to Mn (IV); and (iii) the peaks at the lowest binding energy (around 641 eV) are also associated with Mn (III). So, it seems that Mn(III) and Mn(IV) also coexist on the surface of all BMC-La_x_ samples. Note that the presence of Mn(III) on the surface implies that oxygen vacancies should exist (in order to achieve the neutrality of positive and negative charges on surface), as evidenced in the data for the O 1s transition discussed above. Also, just as observed for the BM-Cex series, as two deconvoluted peaks are found for Mn(III), it seems that different electronic environments exist, probably due to the existence of oxygen vacancies and/or copper species. The Mn(IV)/Mn(III) ratio, included in Table 6 and calculated using the area under the corresponding deconvoluted XPS peaks, reveals that Mn(IV) is the main oxidation state on the surface for BMC and BMC-La_0.1_ as the Mn(IV)/Mn(III) ratio is higher than 1, but as the La percentage increases, the ratio becomes lower than 1; so, Mn(III) seems to be the main oxidation state for BMC-La_0.3_ and BMC-La_0.6_. This trend could be expected due to the increase in the positive charge on the surface caused by the progressive substitution of Ba (II) by La (III), which seems to hinder the oxidation of Mn (III) to Mn (IV); so, Mn(III) is favored by the presence of La, becoming the main oxidation state at high La contents.

On the other hand, three peaks can be fitted for the Cu 2p_3/2_ (Figure 6c) spectra, with binding energies at around 933.0, 934.5, and 940.0–943.0 eV. According to the literature [21,31], these peaks are assigned to Cu(II) with strong (Cu(II)_s_) and weak (Cu(II)_w_) interaction with the perovskite surface for the two former, and to Cu(II) satellite peak for the latest. Upon using the area under the XPS signals for each metal, the Cu/(Ba + Mn + Cu) XPS ratio has been calculated. This value is compared in Table 6 with the nominal one in order to obtain information about the copper distribution. This is because, if the XPS ratio is lower than or similar to the nominal one, this means that copper has been inserted into the perovskite structure; and if it is higher, this implies that copper is being accumulated on the surface [21,31]. According to the data, the distribution of copper is affected by the presence of La, as an increase in the Cu/(Ba + Mn+ Cu) XPS ratio is found. This finding agrees with the XRD data that suggest a lower degree of copper insertion into the perovskite structure (as polytype structure is disfavored); so, the accumulation of copper on the surface increases with the percentage of La. Additionally, the ratio of the copper species with different interactions with perovskite ($\frac{{Cu(II)}_{s}}{{Cu(II)}_{W}}$) has been calculated considering the area under the corresponding deconvoluted peaks, and an increase with lanthanum content was also detected. Thus, even though the copper species have progressively accumulated on the surface, a strong interaction between these surface copper species and the perovskite is favored, presenting BMC-La_0.6_ with the highest proportion of Cu(II)_s_ and BMC with the lowest one. 

**Figure 7:**
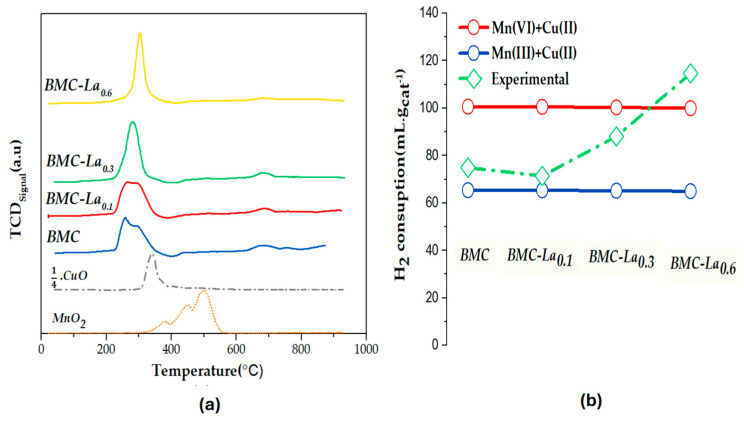
(**a**) H_2_-TPR profiles of BM, BMC-Lax samples, and MnO_2_ and CuO as references; (**b**) H_2_ consumption (mL g^−1^ of catalyst).

H_2_-TPR tests were carried out to examine the reducibility of BMC-Lax samples, and the H_2_ consumption profiles are presented in Figure 7, where they are compared with the corresponding MnO_2_ and CuO (divided by 4 to obtain a similar peak intensity) used as references. As described above for the BM-Cex series, and considering the assignation of the peaks detected in the profile exhibited by the MnO_2_ and CuO references (see previous section for BM-Cex characterization), the three reduction peaks detected in the H_2_-TPR profiles of BMC-Lax samples correspond to (i) the high-intensity peak at the low-temperature region (200–400 °C), to the reduction of Mn(IV) and Mn(III) to Mn(II) and also of Cu(II) to Cu(0) [18,43]; (ii) the second wide and very-low-intensity peak at intermediate temperature region (700–800 °C), to the desorption/reduction of oxygen species; and (iii) the third peak, which is almost inappreciable for this series but usually appears in perovskites at the highest temperature (900–1000 °C), to the reduction of bulk Mn (III). Note that the addition of lanthanum at x > 0.1 provokes an increase in the intensity of the main peak; so, there is an improvement in the reducibility of the BMC-La_0.3_ and BMC-La_0.6_ samples with respect to BMC and BMC-La_0.1_. Additionally, a more-defined peak is featured for the former samples, which, in depth, is more similar to the one shown by CuO reference. This finding agrees with the XPS results (Figure 6c) that reveal a higher proportion of surface copper for these two samples. Focusing our attention on the temperature for the maximum of this peak, it is lower than that observed for the CuO and MnO_2_ references, revealing a synergetic effect between copper and manganese, which was previously found for other copper perovskites [31]. However, the temperature of the maximum is higher in the presence of La, and it increases with the La percentage (from 266 to 279 and 303 °C for BMC-La_0.1_, BMC-La_0.3_, and BMC-La_0.6_ versus 259 °C for BMC), suggesting that the presence of La decreases the Mn-Cu synergetic effect. The area under the hydrogen consumption profiles of Figure 7a was used to determine the experimental hydrogen consumption per gram of catalyst between 200 and 400 °C, which is compared in Figure 7b to the theoretical hydrogen consumption calculated considering the total reduction of manganese and copper—either as Mn(III) + Cu(II) (blue line) or Mn(IV) + Cu(II) (red line). Thus, it appears that the experimental H_2_ consumption is close to the nominal Mn(III) + Cu(II) for BMC and BMC-La_0.1_, and, after increasing the lanthanum percentage in the BMC-La_0.3_ and BMC-La_0.6_ perovskites, it progressively increases until nearing the corresponding nominal Mn(IV) + Cu(II). This trend suggests an increase in the amount of bulk Mn(IV) with the increasing lanthanum percentage in perovskite.

**Figure 8:**
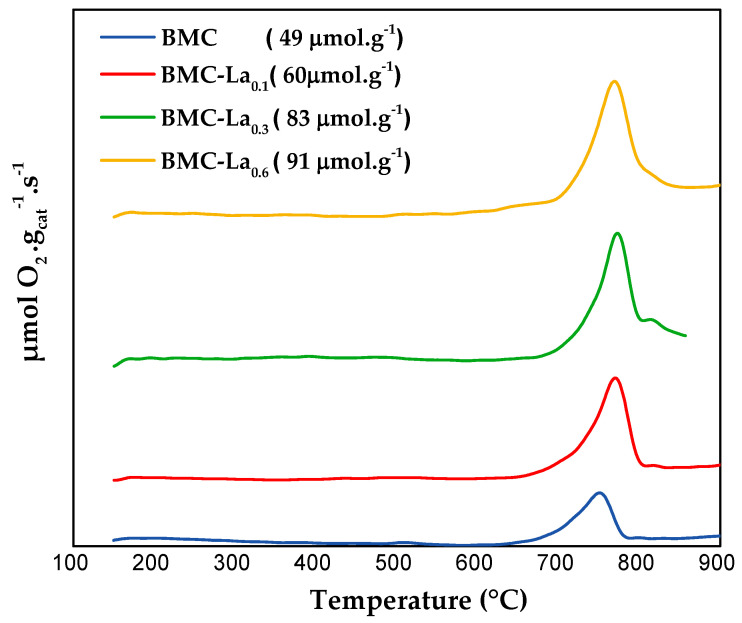
O_2_-TPD profiles of BMC and BMC-Lax samples.

The information about oxygen mobility through the lattice is obtained from the O_2_-TPD tests [1,27,30], with the oxygen-evolved profiles for BMC-Lax being displayed in Figure 8. The samples do not evolve oxygen at temperatures lower than 650 °C, as only a desorption peak between 650 and 900 °C is found, which corresponds to the release of β-O_2_; that is, oxygen comes from the perovskite lattice linked to the reduction of Mn(IV)/Mn(III) and Cu(II) cations [41], which is favored by the presence of bulk oxygen vacancies. In the presence of lanthanum, an increase in the temperature for the maximum is detected (from 753 °C for BMC to 773, 779, and 773 °C for BMC-La_0.1_, BMC-La_0.3_, and BMC-La_0.6_, respectively). The shift towards higher temperatures should be a consequence of a decrease in the Mn-Cu synergetic effect, probably due to the lowering of the amount of Cu inside the structure. Note that the amount of β-O_2_ evolved (calculated using the area under the peak between 650 °C and 900 °C) also increases with the La content from 49 to 91 μmol g^−1^; so, improved oxygen mobility seems to be provoked by the presence of La. This trend agrees with the increase in the reducibility previously observed during H_2_-TPR tests.

### 2.2. Soot Oxidation Tests

**Figure 9:**
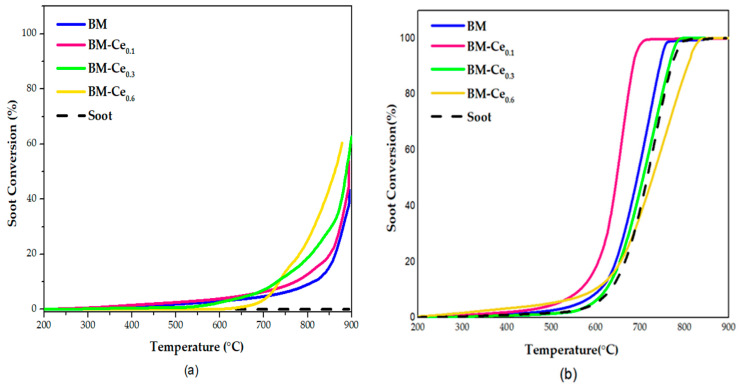
Soot-TPR conversion profiles as a function of the temperature of BM and BM-Ce_x_ catalysts in 100% He (**a**) and in 1% O_2_/He (**b**).

**Figure 10:**
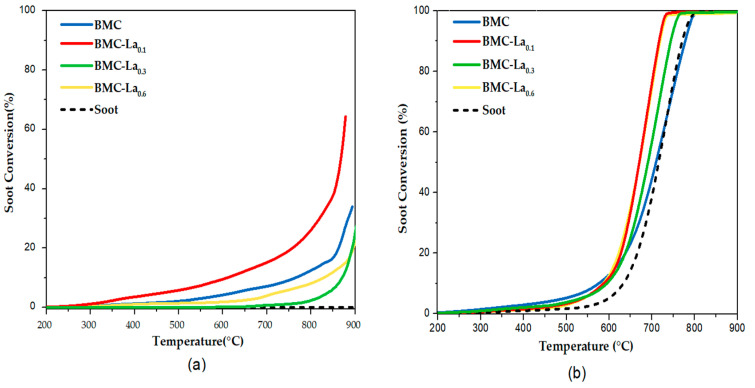
Soot-TPR conversion profiles as a function of temperature of BMC and BMC-La_x_ catalysts in 100% He (**a**) and in 1% O_2_/He (**b**).

The temperature-programmed reaction in the presence of model soot (soot-TPR) was used to assess the catalytic performance of BM-Cex and BMC-Lax perovskites for GDI soot oxidation in the two gaseous mixtures (0% and 1% O_2_ in He) previously used [16]. The soot conversion profiles are presented in Figure 9 for BM-Cex samples and in Figure 10 for BMC-Lax, being selective for CO_2_ (S_CO2_), and the T_10%_ and T_50%_ values (temperatures to achieve 10% and 50% of soot conversion, respectively) presented in Table 7 and Table 8 for the BM-Cex and BMC-Lax series.

Figure 9a depicts the soot conversion profiles in the absence of oxygen for BM-Cex samples, where it is observed that all perovskites catalyze the soot oxidation (if they are compared with the profile obtained in the absence of catalyst), showing a shift of the soot conversion profile towards lower temperatures as the Ce content increases. In the presence of perovskites, the reaction seems to occur with the oxygen evolved from the samples, which is mainly β-O_2_, as the soot conversion is mainly promoted at temperatures higher than 650 °C (see Figure 4). Consequently, the temperature for soot conversion directly depends on the temperature at which the β-O_2_ starts to be evolved from the samples; that is, at T > 700 °C. From the BM-Cex series, BM-Ce_0.6_ is the most-active catalyst as it features the highest soot conversion values at T > 700 °C. T_10%_ data in Table 7 evidence that BM-Ce_0.3_ and BM-Ce_0.6_ perovskites present the lowest T_10%_ values among the BM-Cex samples. Note that these two perovskites evolve a higher amount of oxygen during the O_2_-TPD tests than the BM-Ce_0.1_ and BM raw samples, probably due to the contribution of Ce(IV)/Ce(III) reduction as they present a higher amount of Ce(IV), both on the surface (as revealed via XPS) and in the bulk (due to the presence of CeO_2_, as identified via XRD), than BM-Ce_0.1_ and BM.

Figure 9b and the data in Table 7 present the soot-TPR profiles and the S_CO2_, T_50%_, and T_10%_ data for BM-Cex samples in a 1% O_2_ atmosphere. In the presence of oxygen, it seems that only the introduction of the lowest amount of Ce (BM-Ce_0.1_) allows us to improve the performance of the BM raw sample in terms of T_10%_, T_50%_, and S_CO2_ as, after increasing the Ce percentage, the role of the catalysts seems to be worse, as T_10%_ and T_50%_ values increase and S_CO2_ decreases in the BM-Ce_0.3_ and BM-Ce_0.6_. In fact, BM-Ce_0.6_ presents a significantly low catalytic activity, remaining so even under the uncatalyzed test at high temperatures. According to characterization data, this trend seems being related to (i) the lower amount of oxygen surface vacancies as cerium content increases (see XPS results in Table 3); (ii) the significant decrease in the amount of Mn(IV) on the surface (XPS) and in the bulk (H_2_-TPR); (iii) a lower reducibility (H_2_-TPR) for BM-Ce_0.3_ and BM-Ce_0.6_ with respect to BM-Ce_0.1_; and (iv) the increasing amount of CeO_2_ (see XRD patterns in Figure 1), as it has been reported that this phase presents a poor catalytic activity for soot oxidation [46]. In conclusion, the increase in cerium content in the BM-Cex samples does not improve the catalytic performance of soot oxidation in 1% O_2_/H, as shown by BM-Ce_0.1_; consequently, this is the best catalyst. Thus, it seems that the similar amount of surface oxygen vacancies to BM raw perovskite and the highest proportion of Mn(IV) and Ce(IV) among all BM-Ce_x_ samples make BM-Ce_0.1_ the best catalyst.

Regarding the soot conversion profiles presented in Figure 10, and the S_CO2_, T_10%,_ and T_50%_ data compiled in Table 8 for BMC-La_x_ samples, it seems that, independently of the presence of oxygen in the reaction atmosphere, the increase in the lanthanum content does not improve the catalytic performance of BMC-La_0.1_, even though BMC-La_0.3_ and BMC-La_0.6_ are able to catalyze the soot oxidation reaction, because they present lower T_10%_ and T_50%_ values and a higher S_CO2_ than that observed for the uncatalyzed reaction. Based on the characterization results, it seems that this trend is caused by the decrease in the amount of Mn(IV) with respect to BMC-La_0.1,_ which does not seem to be compensated by the increase in the amount of surface Cu(II).

In summary, the increase in the amount of Ce (from x = 0.1 to x = 0.6) only improves the catalytic performance of the BM raw sample for soot oxidation if the oxygen coming from perovskite is uniquely available for soot oxidation, which seems to be due to the contribution of the Ce(IV)/Ce(III) redox pair to the lattice oxygen (β-O_2_) generation.

Finally, by comparing the catalytic performance in soot oxidation of the two tested series, BM-Cex and BMC-Lax, and in the two conditions used, the following conclusions are reached:

(i) In the absence of oxygen in the reaction atmosphere (100% He), BMC-La_0.1_ is the best catalyst, as copper is also able to catalyze the soot oxidation [47].
(ii) If oxygen is present in the reaction atmosphere (1% O_2_/He), BM-Ce_0.1_ is the most active catalyst as it presents a higher proportion of Mn(IV) than BMC-La_0.1_.

Thus, it can be concluded that the addition of an amount of Ce or La dopant higher than that corresponding to x = 0.1 in Ba_1−x_Ce_x_MnO_3_ and Ba_1−x_La_x_Cu_0.3_Mn_0.7_O_3_ does not allow us to improve the catalytic performance of BM-Ce_0.1_ and BMC-La_0.1_ for soot oxidation in the conditions tested.

## 3. Materials and Methods

### 3.1. Synthesis

The sol–gel process adjusted to aqueous media [31,46,48,49] was used for the synthesis of Ba_1−x_Ce_x_MnO_3_ and Ba_1−x_La_x_Mn_0.7_Cu_0.3_O_3_ (x = 0, 0.1, 0.3, 0.6) perovskites. Barium acetate (Ba(CH_3_COO)_2_, Sigma-Aldrich, St. Louis, MO, USA, 99.0% purity), lanthanum nitrate hydrate (La(NO_3_)_3_·H_2_O, Sigma-Aldrich, 99.0% purity), cerium(III) nitrate hexahydrate (Ce(NO_3_)_3_·6H_2_O, Sigma-Aldrich, 99.0% purity), copper(II) nitrate trihydrate (Cu(NO_3_)_2_·3H_2_O, Panreac, Castellar del Vallès, Spain, 99.0% purity), and manganese(II) nitrate tetrahydrate (Mn(NO_3_)_2_·4H_2_O, Sigma-Aldrich, 99.0% purity) were employed as precursors. In order to avoid the precipitation of precursors, citric acid (C_6_H_8_O_7_, Sigma-Aldrich, 98.5% purity) was used as a complexing and chelating agent (with a molar ratio citric acid/Ba of 2). The procedure begins with a heating of 40 mL of a citric acid solution to 60 °C while continuously stirring. After adding the metal precursors, the temperature is raised to 65 °C for five hours to allow for the gel formation. During the whole synthesis, a 30% ammonia solution (from Panreac, Castellar del Vallès, Spain, 99.0% purity) allowed us to maintain the pH at 8.5. At the end of the process, the gel was dried for 48 h at 90 °C, and finally, the solid was calcined for 6 h at 850 °C.

### 3.2. Characterization

The perovskite samples were characterized using the following techniques:

- ICP-OES to ascertain the actual percentage of elements: To carry out this analysis, 10 mg of sample were dissolved in 5 mL of aqua regia diluted in 10 mL of distilled water. The analysis was performed using an Optimal 4300 DV Perkin-Elmer instrument (Waltham, MA, USA).
- N_2_ adsorption at −196 °C, employing an Autosorb-6B device from Quanta Chrome (Anton Paar Austria GmbH, Graz, Austria) to measure the BET surface area.
- XRD to determine the crystalline structure: The X-ray patterns were captured using a Bruker D8-Advance device (Billerica, MA, USA) using the Cu K_α_ radiation (1.4506 Å) and a step rate of 0.4° min^−1^ between 20° and 80° 2θ angles. On the other hand, the Williamson–Hall method has been employed as it allows for more accuracy in the calculation of the average crystal size because it discards the contribution of the lattice strain to the full width at half maximum (FWHM) of the XRD peaks [22]. Finally, XRD refinement was performed to determine the percentage of the different crystalline phases in the sample [50] by using the HighScore Plus software (Malvern Panalytical B.V. Almelo, The Nertherlands, 4.9 (4.9.0.27512) version).
- XPS to evaluate the surface composition: An Al K_α_ (1486.7 eV) radiation source and a Thermo-Scientific K-Alpha photoelectron spectrometer (Thermo-Scientific, Waltham, MA, USA) were used, and, to obtain the XPS spectra, the pressure within the analysis chamber was maintained at 5 × 10^−10^ mbar. The spectrometer’s peak-fit software (Thermo Avantage v5.9929) was used to determine the binding energy (BE) and kinetic energy (KE) values. The C 1s transition was set at 284.6 eV.
- H_2_-TPR for assessing the reducibility of samples. To develop the tests, 30 mg of sample were heated at 10 °C min^−1^ from 25 °C to 1000 °C and 40 mL min^−1^ of a gaseous mixture consisting of 5% H_2_/Ar were used. A CuO reference sample, which is reduced to Cu^0^, was employed to quantify the H_2_ consumption. The tests were carried out in a Pulse Chemisorb 2705 (from Micromeritics, Norcross, GA, USA) outfitted with a Thermal Conductivity Detector (TCD).
- O_2_-TPD to estimate the oxygen evolved from samples, which informs about the mobility of oxygen. Using a Thermal Gravimetric-Mass Spectrometer equipment (TG-MS, Q-600-TA, and Thermostar from Balzers Instruments, (Balzers, Liechtenstein), 16 mg of sample was heated in a helium gas flow (100 mL min^−1^) at a rate of 10 °C min^−1^ to 950 °C. In order to remove the moisture, each sample was heated to 150 °C for one hour before the tests. The 18, 28, 32, and 44 *m*/*z* signals were recorded to follow the emission of H_2_O, CO, O_2_, and CO_2_. The quantification of the amount of oxygen evolved was performed also using a CuO reference sample, which is reduced to Cu_2_O.

### 3.3. Catalyst Activity

The TG-MS system used for O_2_-TPD was also employed to carry out the soot oxidation tests that were conducted, replicating two representative scenarios for the GDI engine exhaust: (i) 100% He, which mimics standard GDI engine operations; and (ii) 1% O_2_/He, which represents the “fuel cuts” in the GDI engine exhaust conditions [16,17,48]. To develop the tests, a 1% O_2_/He mixture (100 mL min^−1^) was used to preheat (at 150 °C, 1 h) 16 mg of a catalyst and soot mixture (soot: catalyst ratio of 1:8, using Printex-U as model soot in loose contact mode); and after, the temperature was raised to 900 °C at 10 °C min^−1^ (soot-TPR).

The soot conversion and the selectivity to CO_2_ percentages were calculated using the following Equations:

(1) $$Soot\;conversion\left(\%\right)=\frac{\sum_{0}^{t}co_{2}+co}{\sum_{0}^{\mathrm{final}}(co_{2}+co)}\times 100,$$

(2) $$Selectivity\;to\;CO_{2}=\frac{CO_{2\;total}}{{\left(CO_{2}+CO\right)}_{total}}\times 100,$$

where Σ_0_^t^ CO_2_ + CO is the amount of CO_2_ and CO evolved at time *t*, while Σ_0_^final^ CO_2_ + CO is the total amount of CO + CO_2_ evolved during the experiment, coming from the oxidation of the total amount of soot.

## 4. Conclusions

In this paper, Ba_1−x_Ce_x_MnO_3_ (BM-Cex) and Ba_1−x_La_x_Mn_0.7_Cu_0.3_O_3_ (BMC-Lax) perovskite-type mixed oxides (x = 0, 0.1, 0.3, 0.6) were synthesized, characterized, and tested as catalysts for soot oxidation in simulated GDI exhaust conditions. The discussion of the results suggests the following conclusions:

- Based on the characterization of samples, the following conclusions can be drawn:
  (i) For the BM-Cex series: (i.1) as the percentage of Ce increases, the hexagonal perovskite structure is progressively replaced by CeO_2_ crystalline phase, which is the main one for BM-Ce_0.6_; (i.2) Mn(IV) is the main oxidation state on the surface for BM and BM-Ce_0.1_, but it is Mn(III) for BM-Ce_0.3_, while for BM-Ce_0.6_, almost-similar amounts of Mn(III) and Mn(IV) are present; (i.3) Ce(III) and Ce(IV) coexist on the surface of all BM-Cex samples, and a considerable increase in the surface Ce(IV) proportion is detected from BM-Ce_0.1_ to BM-Ce_0.6_; (i.4) in the presence of Ce, the reduction of Mn/Ce takes place at lower temperatures due to the synergetic effect between Mn and Ce; and (i.5) the oxygen mobility through the perovskite lattice increases for Ce samples (due to the contribution of Ce(IV)/Ce(III) redox pair), and all of them evolve β-O_2_, but only BM-Ce_0.1_ generates a low amount of α′-O_2._
  (ii) For the BMC-Lax series: (ii.1) as the percentage of lanthanum increases, the intensity of XRD peaks corresponding to the BaMnO_3_ polytype structure decreases in favor of an increase in the intensity of the peaks corresponding to hexagonal 2H-BaMnO_3_ and trigonal La_0.93_MnO_3_ perovskite structures, with the latter being the main phase for BMC-La_0.6_; (ii.2) the amount of surface oxygen vacancies seems not to be sensitive to the increase in the La amount in samples; (ii.3) Mn (III) and Mn (IV) coexist on the surface and in the bulk; however, on the surface, Mn(III) increases with the La content; while in the bulk, Mn(IV) is favored as La content increases; (i.4) the accumulation of Cu (II) on the surface increases with the percentage of La; (ii.5) an increase in the reducibility of BMC-La_0.3_ and BMC-La_0.6_ samples with respect to BMC and BMC-La_0.1_ is found; and (ii.6) the oxygen mobility increases with the percentage of La.
- Based on the analysis of the catalytic performance for soot oxidation in the two conditions tested: (i) in the absence of oxygen in the reaction atmosphere (100% He), BMC-La_0.1_ is the best catalyst as copper is also able to catalyze the soot oxidation; (ii) if oxygen is present in the reaction atmosphere (1% O_2_/He), BM-Ce_0.1_ is the most-active catalyst as it presents a higher proportion of Mn(IV) than BMC-La_0.1_. Thus, the addition of an amount of Ce or La dopant higher than that corresponding to x = 0.1 in Ba_1−x_Ce_x_MnO_3_ and Ba_1−x_La_x_Cu_0.3_Mn_0.7_O_3_ does not allow us to improve the catalytic performance of BM-Ce_0.1_ and BMC-La_0.1_ for soot oxidation in the tested conditions.

## Figures and Tables

**Table 1:** BET surface area and cerium content of BM-Cex samples.

Nomenclature	Sample	BET (m^2^ g^−1^)	Ce (wt%)
BM	BaMnO_3_	3	-
BM-Ce_0.1_	Ba_0.9_Ce_0.1_MnO_3_	10	1.3
BM-Ce_0.3_	Ba_0.7_Ce_0.3_MnO_3_	7	3.1
BM-Ce_0.6_	Ba_0.4_Ce_0.6_MnO_3_	3	6.0

**Table 2:** XRD data of BM-Cex samples.

Sample	Hexagonal 2H-BaMnO_3_ (wt%)	CeO_2_ (wt%)	MnO_2_ (wt%)	Intensity (a.u) ^a^	Average Crystal Size (nm) ^b^	BaMnO_3_ Cell Parameters (Å) ^c^	
						a	c
BM	94	-	6	1154	46.0	5.7	4.9
BM-Ce_0.1_	76	14	10	1913	22.0	5.5	5.0
BM-Ce_0.3_	53	29	18	720	14.4	5.7	4.8
BM-Ce_0.6_	29	71	-	1708	33	5.7	4.8

^a^ Corresponding to hexagonal- 2H-BaMnO_3_, except for BM-Ce_0.6_, which corresponds to CeO_2_. ^b^ Calculated using the Williamson–Hall method for the XRD peaks of hexagonal 2H-BaMnO_3_ (around 2θ = 26°, 32°, and 41°), except for BM-Ce_0.6_, which corresponds to CeO_2_ (around 2θ = 29°, 31° and 33°) due to the low intensity of the hexagonal perovskite peaks. ^c^ Calculated using the main perovskite XRD peak of the BaMnO_3_ hexagonal structure (2θ = 32°).

**Table 3:** XPS data of BM-Cex samples.

Sample	BE Max Mn(III) (eV)	BE Max Mn(IV) (eV)	BE Max O_L_ (eV)	BE Max O_ads_ (eV)	$\frac{O_{L}}{Ba+Ce+Mn}$	$\frac{Ce\left(IV\right)}{Ce\left(III\right)}$ (CeO_2_) 1.8	$\frac{Mn(IV)}{Mn(III)}$
BM	641.4	642.3	528.9	530.8	1.0	---	1.7
BM-Ce_0.1_	641.4	642.4	529.0	530.7	1.0	1.1	1.4
BM-Ce_0.3_	641.2	642.2	528.9	530.4	1.1	1.4	0.5
BM-Ce_0.6_	641.1	642.2	528.8	530.1	1.2	1.6	0.9

**Table 4:** BET surface area and lanthanum and copper content of BMC-Lax samples.

Nomenclature	Molecular Formula	S _BET_ (m^2^ g^−1^)	Metal Content (wt%)	
			La	Cu
BMC	BaMn_0.7_Cu_0.3_O_3_	3.0	-	8.0
BMC-La_0.1_	Ba_0.9_La_0.1_Mn_0.7_Cu_0.3_O_3_	7.0	5.4	9.8
BMC-La_0.3_	Ba_0.7_La_0.3_Mn_0.7_Cu_0.3_O_3_	9.0	11.0	7.8
BMC-La_0.6_	Ba_0.4_La_0.6_Mn_0.7_Cu_0.3_O_3_	4.0	24.0	7.1

**Table 5:** XRD data.

Sample	Hexagonal 2H-BaMnO_3_ (wt%)	Polytype BaMnO_3_ (wt%)	Trigonal La_0.93_MnO_3_ (wt%)	BaMn_2_O_3_ (wt%)	Average Crystal Size (nm) ^a^	Lattice Parameters (Å) ^b^	
						a	c
BMC	-	100	-		30.7	5.8	4.3
BMC-La_0.1_	4	86	-	10	18.6	5.7	4.3
BMC-La_0.3_	1	47	52	-	21.9	5.5	13.3
BMC-La_0.6_	7	12	81	-	27.6	5.5	13.3

^a^ Calculated using the Williamson–Hall method, employing polytype BaMnO_3_ structure peaks (around 2θ = 27°, 31°, and 42°) for BMC and BMC-La_0.1_, and La_0.93_MnO_3_ trigonal structure peaks (around 2θ = 33°, 47°, and 58°) for BMC-La_0.3_ and BMC-La_0.6_ since the polytype peaks present a low intensity. ^b^ Calculated using the XRD main peak of the polytype structure (around 2θ = 31°) for BMC and BMC-La_0.1_, and the La_0.93_MnO_3_ trigonal structure main peak (around 2θ = 33°) for BMC-La_0.3_ and BMC-La_0.6_.

**Table 6:** XPS data of BMC and BMC-Lax.

Catalyst	$\frac{O_{L}}{Ba+La+Mn+Cu}$	$\frac{Mn(IV)}{Mn(III)}$	$\frac{Cu}{Ba+La+Cu+Mn}$	$\frac{{Cu(II)}_{s}}{{Cu(II)}_{W}}$
	Nominal = 1.5		Nominal = 0.15	
BMC	0.8	1.3	0.09	2.2
BMC-La_0.1_	0.9	1.1	0.10	4.7
BMC-La_0.3_	0.9	0.8	0.11	9.9
BMC-La_0.6_	0.9	0.7	0.13	11.9

**Table 7:** T_10%_, T_50%_, and S_CO2_ for soot oxidation in the two tested atmospheres for the uncatalyzed system: BM and BM-Cex.

Sample	1% O_2_/He			100% He
	S_CO2_ (%)	T_50%_ (°C)	T_10%_ (°C)	T_10%_ (°C)
Soot (uncatalyzed)	44	714	631	-
BM	73	710	590	813
BM-Ce_0.1_	90	641	548	772
BM-Ce_0.3_	42	712	597	730
BM-Ce_0.6_	32	728	622	730

**Table 8:** T_10%_, T_50%_, and S_CO2_ for soot oxidation in the two tested atmospheres of BMC-La_x_ catalysts.

Catalysts	1% O_2_/He			100% He
	S_CO2_ (%)	T_50%_ (°C)	T_10%_ (°C)	T_10%_ (°C)
Uncatalyzed	44	714	631	-
BMC	70	709	599	879
BMC-La_0.1_	94	671	588	611
BMC-La_0.3_	41	689	593	869
BMC-La_0.6_	61	671	585	829

## Data Availability

Data are contained within the article.
